# Teaching Music

**Published:** 1879-09

**Authors:** 


### TEACHING MUSIC.

Some few years since my own mother thought something could be done in the way of teaching children to sing by note, and to sing independently. So she invited about 40 of the village children to meet her every Saturday afternoon for an hour. They came, and she began at the foundation. By the aid of a blackboard she took the class through the primary steps of a musical education. In a few weeks they were able to take up such simple tunes as Boylston and Naomi. Their joy at being able to sing even these tunes, at sight, was unbounded. They became very enthusiastic, and Saturday playtime was gladly given up for music. In the course of one summer she led this class so far that they could take up readily common psalm-tunes, singing them by note, getting the names of the notes, the time, and the tune correctly. The *pons asinorum* of vocal music is the interval. When one can recognise and strike an interval, all else comes easily.

